# Entorhinal Cortex and Persistent Olfactory Loss in COVID-19 Patients: A Neuroanatomical Hypothesis. Comment on Fiorentino et al. Correlations between Persistent Olfactory and Semantic Memory Disorders after SARS-CoV-2 Infection. *Brain Sci.* 2022, *12*, 714

**DOI:** 10.3390/brainsci12070850

**Published:** 2022-06-29

**Authors:** Pietro De Luca, Pasquale Marra, Ignazio La Mantia, Francesco Antonio Salzano, Angelo Camaioni, Arianna Di Stadio

**Affiliations:** 1 Department of Medicine, Surgery and Dentistry, University of Salerno, 84084 Salerno, Italy; pasquale.marra.m178@gmail.com (P.M.); frsalzano@unisa.it (F.A.S.); 2 Otolaryngology Department, San Giovanni-Addolorata Hospital, 00133 Rome, Italy; acamaioni@hsangiovanni.roma.it; 3 Otolaryngology Department, University of Catania, 95123 Catania, Italy; ilamantia@unict.it

Recently, Fiorentino et al. [1] published a paper in which they investigated the association between semantic memory impairment in long COVID-19 patients and persistent olfactory disturbances. Semantic memory was impaired in 20% of these patients, especially the youngest patients (19–39 age-group), and the olfactory threshold score had the only significant correlation with semantic memory scores. This recent study confirmed the data from Di Stadio et al. [2], who found a correlation between the presence of memory alteration and the highest severity of quantity and quality smell alterations. Both articles underlined the importance of memory functions in the smell process and clinically evidenced that the alteration of memory had an impact on odor recognition memory, which begs the question, what may be the link between memory and smell?

We want to discuss an intriguing hypothesis to explain the clinical data observed by these two authors. 

Currently, it is well-known that COVID-19 causes systemic inflammation and brain neuro-inflammation [3]; this neuro-inflammation could be caused by direct inflammation of the brain tissue [3,4] or be related to systemic alterations caused by COVID-19, such as cytokines storm and micro-/thrombotic events [5]. The inflammation, starting from the olfactory bulbs, spreads to other areas of the brain and causes several symptoms [5], including forgetfulness, short-term memory, mental clouding, lack of concentration, and attention deficits [6,7,8,9]. 

From a clinical point of view, neuro-inflammation could explain both mental clouding (brain fog) and headache [2] and why their presence was correlated with worse quantitative smell alterations compared to patients without neurological symptoms. It is also important to underline that odor perception has a hedonic component [10], and odors can both stimulate memory and emotions (positive and negative), so smell alterations may also impact memory functions. 

The entorhinal cortex (EC) might explain the link between smell alterations and memory impairment that has been observed in patients with a persistence of these two symptoms (long COVID). The EC (Brodman’s area 28) is a brain area located in the medial temporal bone in the rostral parahippocampal gyrus [11]; it represents the major interface between the hippocampus and sensory cortices and forms the nodal point in cortico-hippocampal circuits [12,13].

The EC receives inputs from olfactory bulbs and the piriform cortex, and projects back to these areas, modulating odor-evoked activity in the piriform cortex [14]; the lateral EC (LEC) in particular provides a highly odor-specific memory feedback to the olfactory cortex. The study by Chapuis et al. [15] provided enough data to confirm how LEC modulates piriform cortical activity and fine-odor discrimination, also explaining that the neuropathological changes in the EC could contribute to early olfactory deficit in Alzheimer’s disease (AD) [16] (Figure 1).

Additionally, the EC is in close contact with the hippocampus, which has both mnemonic and emotional functions; therefore, for contiguity, the neuro-inflammation might spread from EC to this structure, causing a worsening of the memory function and the alteration of moods with an altered perception of the odors. 

The EC is the first area affected by Alzheimer’s disease (AD); the area is smaller in patients with AD than those affected by normal age-related cognitive impairment [17]. Given that SARS-CoV-2 infection causes neuro-inflammation, the impact on the EC may be responsible for the memory alterations observed post-COVID. Moreover, LEC computes and transfers olfactory information from the olfactory bulb to the hippocampus [18]. Neuro-inflammation in the EC causes an alteration in odor processing with an indirect impact on the hippocampus function; in this way, both olfactory and memory functions could be impaired. We speculate that the EC might be the key area to explain not only the presence of olfactory and memory problems in patients with long COVID [2], but also the correlation between mental clouding and odor perception.

This hypothesis can be confirmed by studying the results of Douadud et al. [19]; the authors used magnetic resonance imaging (MRI) to analyze patients affected by SARS-CoV-2 who suffered from an alteration of memory functions and identified markers of tissue damage in regions functionally connected to the primary olfactory cortex, with reduced grey matter thickness and reduced tissues contrast in the orbitofrontal cortex and parahippocampal gyrus. Considering that many neuronal connections from and to the olfactory bulb involve regions of the EC, the piriform cortex, orbitofronal areas, and parahippocampal gyrus, the alteration observed by Douadud might be indirectly linked to malfunction of the EC [20].

Based on MRI studies, which confirmed an EC atrophy preceding hippocampal atrophy in AD [21], and the observed clinical results [1,2], the EC might be the link between olfactory loss and the reduction in the memory function. In fact, it seems that even minimal EC atrophy might cause a greater impairment in cognitive test performance, even greater than in patients with atrophy of the hippocampus [22].

Specific functional MRI (fMRI) studies should be performed to evaluate the activity of the EC in long COVID patients suffering from persistent olfactory and neurocognitive symptoms to confirm our speculation. 

In addition, some predisposed patients suffered from persistent neuro-inflammation in COVID-19 [23], and untreated neuro-inflammation can lead to neurodegeneration (olfactory bulbs, frontal cortex, EC, etc.). Due to this, and given the potential impact of neuro-inflammation caused by COVID-19 infection on memory, future research exploring the use of systemic steroids or anti-neuroinflammations molecules [24] may help inform clinical treatment options.

## Figures and Tables

**Figure 1:**
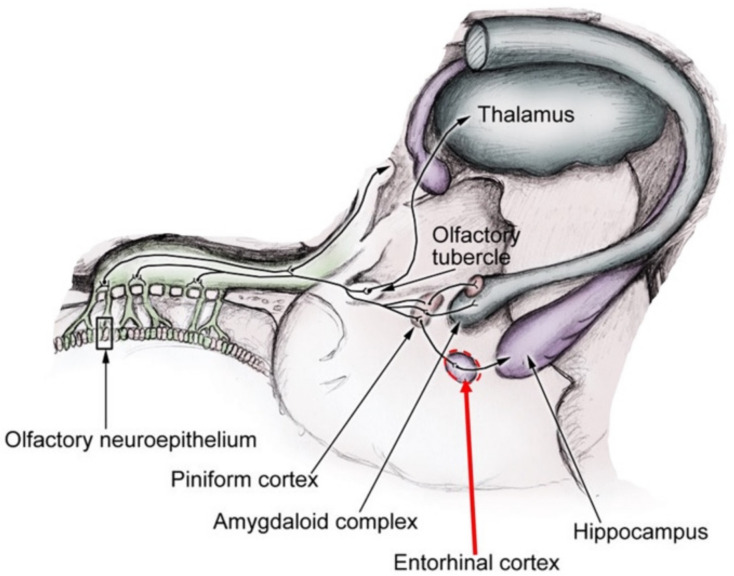
Anatomical visualization of olfactory pathways and mnemonic connections, with a central role of the entorhinal cortex.
